# Current Data on the Role of Amino Acids in the Management of Obesity in Children and Adolescents

**DOI:** 10.3390/ijms26157129

**Published:** 2025-07-24

**Authors:** Diana Zamosteanu, Nina Filip, Laura Mihaela Trandafir, Elena Ţarcă, Mihaela Pertea, Gabriela Bordeianu, Jana Bernic, Anne Marie Heredea, Elena Cojocaru

**Affiliations:** 1 Department of Morphofunctional Sciences I—Pathology, Faculty of Medicine, “Grigore T. Popa” University of Medicine and Pharmacy, 700115 Iasi, Romania; zamosteanu.diana@d.umfiasi.ro (D.Z.); elena2.cojocaru@umfiasi.ro (E.C.); 2 Department of Morphofunctional Sciences II—Biochemistry, Faculty of Medicine, “Grigore T. Popa” University of Medicine and Pharmacy, 700115 Iasi, Romania; gabriela.bordeianu@umfiasi.ro; 3 Department of Mother and Child—Pediatrics, Faculty of Medicine, “Grigore T. Popa” University of Medicine and Pharmacy, 700115 Iasi, Romania; laura.trandafir@umfiasi.ro; 4 Department of Surgery II—Pediatric and Orthopedic Surgery, Faculty of Medicine, “Grigore T. Popa” University of Medicine and Pharmacy, 700115 Iasi, Romania; tarca.elena@umfiasi.ro; 5 Department of Plastic Surgery and Reconstructive, Faculty of Medicine, “Grigore T. Popa” University of Medicine and Pharmacy, 700115 Iasi, Romania; mihaela.pertea@umfiasi.ro; 6 Discipline of Pediatric Surgery, “Nicolae Testemitanu” State University of Medicine and Pharmacy, MD-2001 Chisinau, Moldova; jana.bernic@usmf.md; 7 Department of Prosthetic Technology and Dental Materials, “Victor Babeș” University of Medicine and Pharmacy, 300041 Timișoara, Romania; anneheredea@gmail.com; 8 Research Center in Dental Medicine Using Conventional and Alternative Technologies, “Victor Babeș” University of Medicine and Pharmacy, 300041 Timișoara, Romania

**Keywords:** amino acids, childhood obesity, metabolic dysfunction, diabetes, insulin resistance

## Abstract

Childhood obesity is a major global health problem, and its management involves a multidisciplinary approach that includes lifestyle changes, dietary interventions, and the use of dietary supplements. In this review, we summarize current findings on the role of amino acids in pediatric obesity, with a particular focus on their involvement in metabolic pathways and weight regulation. The involvement of branched-chain and aromatic amino acids in the pathophysiology and potential management of pediatric obesity is highlighted in recent studies. Both experimental and clinical studies have shown that obese children often exhibit altered plasma amino acid profiles, including increased levels of leucine, isoleucine, valine, phenylalanine, and tyrosine, as well as decreased levels of glycine and serine. These imbalances are correlated with insulin resistance, inflammation, and early metabolic dysfunction. One of the mechanisms through which branched-chain amino acids can promote insulin resistance is the activation of the mammalian target of rapamycin (mTOR) signaling pathway. Metabolomic profiling has demonstrated the potential of specific amino acid patterns to predict obesity-related complications before they become clinically evident. Early identification of these biomarkers could be of great help for individualized interventions. Although clinical studies indicate that changes in dietary amino acids could lead to modest weight loss, improved metabolic profiles, and increased satiety, further studies are needed to establish standardized recommendations.

## 1. Introduction

As of 2020, an estimated 39 million children under the age of five were overweight or obese. The rise in body mass index (BMI) in children and adolescents across countries has been a constant concern for health professionals [1,2]. Between 1975 and 2016, global trends showed that obesity and overweight rates increased most in low- and middle-income countries [3]. Almost one in five children and adolescents (aged 6–19 years) were overweight or obese in 2016 [4]. The global trend in childhood obesity has shown a steady increase in BMI in children and adolescents over four decades in high-income countries in Europe and North America, as well as in some low- and middle-income countries [5]. Obesity in early life is of concern because of its health consequences and its influence on later life. There is an increased risk of becoming an obese adult if individuals are obese at the age of 2, 5, or 10 years. The development of obesity is closely associated with the development of metabolic disorders, such as insulin resistance, type 2 diabetes, and the manifestation of adverse cardiometabolic disease signs. There is a threefold increase in the prevalence of metabolic syndrome in obese children compared with non-obese children. Being overweight at a young age poses a greater health risk than being overweight in adulthood [6,7]. Existing studies show that a relatively higher risk of developing obesity is associated with children who have poor eating habits, such as a higher intake of energy-dense foods and excessive food consumption [8,9]. Currently, the prevalence of diet-related metabolic disorders, such as obesity and glucose intolerance, is increasing. Obesity has become a real public health challenge in most countries, including developing countries, where there is a convergence between undernutrition and overnutrition. In Asian countries, a relatively lower body weight is acceptable. However, at similar levels of adiposity, Asian children, compared with children from other ethnic backgrounds, have a higher risk of dyslipidemia and insulin resistance [10]. Dietary factors are the most important factors associated with childhood obesity, and the prevalence of nutrition-related diseases has led to the prioritization of healthy diets [11]. Dietary components, such as energy-dense foods, sugar-sweetened beverages, and processed food consumption patterns, are discussed and evaluated as modifiable risk factors associated with obesity in children and adolescents.

## 2. Brief Overview of Amino Acids and Their Metabolic Regulation

**Figure 1:**
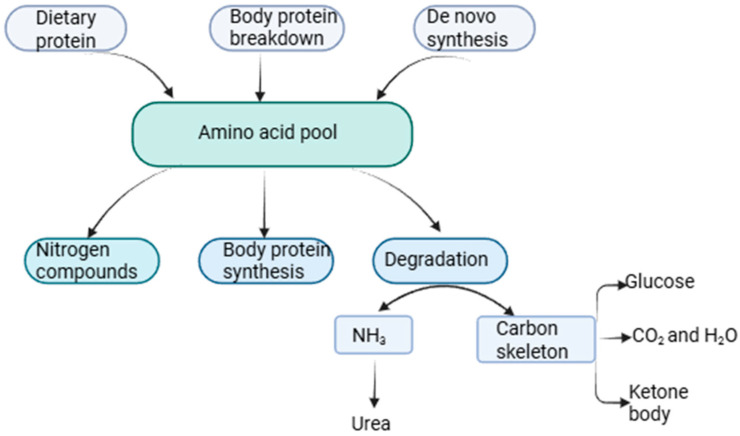
The amino acid pool contains amino acids derived from dietary proteins, body protein breakdown, and de novo synthesis. Created in BioRender.com. Nina Filip (2025) https://app.biorender.com/illustrations/67fdf01102e7dbc88f5082c1?slideId=59479ee2-5643-48f7-a0b4-678bd9eb4e34 (accessed on 18 June 2025).

Amino acids are compounds involved in various metabolic processes in the body, including protein synthesis, energy production, nutrient sensing, immune response, and communication between organs. The human body can obtain amino acids through the digestion and absorption of dietary proteins, through tissue decomposition, and through endogenous synthesis. There are 20 proteinogenic amino acids, 9 of which cannot be synthesized from other compounds and must be supplied through dietary intake; these are called essential amino acids [12]. Amino acids ingested from food, in addition to being used for the synthesis of proteins and other compounds, can be oxidized to urea and carbon dioxide as energy sources through oxidative pathways (Figure 1). In addition to being the essential components of proteins, amino acids are involved in metabolic pathways that maintain cell growth, metabolism, and immunity [13].

A pivotal role in controlling protein synthesis and cellular growth is played by the mammalian target of rapamycin (mTOR) signaling pathway [14]. This pathway comprises two distinct complexes: rapamycin-sensitive complex 1 (mTORC1) and rapamycin-insensitive complex 2 (mTORC2). The activation of mTORC1 is stimulated by specific amino acids such as glutamine (Gln), arginine (Arg), and leucine (Leu). Beyond protein synthesis, amino acids influence other metabolic processes. Amino acid alanine (Ala) plays a regulatory role within the glucose metabolism through the so-called alanine kinase—an umbrella term for some complex modulating effects [13]. Ala modulates the AMP-activated protein kinase (AMPK), which acts as a cellular energy sensor being activated in the presence of a high AMP/ATP ratio, a situation indicative of energy stress. Adachi et al. linked Ala metabolism to a consumption of Krebs cycle intermediary compounds such as alpha-ketoglutarate, thus decreasing ATP production [15]. Furthermore, Ala may also promote urea synthesis, which also consumes ATP; both mechanisms tend to increase the AMP/ATP ratio, thus activating the AMPK. Activated AMPK has an inhibitory effect on the mTOR signaling pathway, thus increasing cellular glucose uptake, glycolysis, and lipolysis and decreasing hepatic glucose production, with the final effect of restoring glucose homeostasis. Arg modulates the urea cycle by acting as an allosteric activator of *N*-acetyl glutamate synthetase [13]. Amino acids are also important in immune regulation. During T cell activation, there is an increased expression of various amino acid transporters, notably SLC7A5. Loss of this transporter disrupts amino acid uptake, triggering changes in the mTOR pathway and increasing expression of MYC, a transcription factor crucial for cell proliferation. Moreover, deprivation of tryptophan (Trp) and Arg halts T cells from progressing into the S phase of the cell cycle, highlighting their importance in T cell proliferation. Similarly, a deficiency in Leu and isoleucine (Ile) leads to T cells arresting at the S-G1 phase, preventing further division and ultimately resulting in cell death [13].

Most studies have been conducted on the branched-chain amino acids (BCAAs)—Leu, Ile, and Val—which have increased concentrations in obese children and adolescents. High circulating levels of BCAAs are associated with insulin resistance, adiposity, and increased risk of type 2 diabetes [16]. Leu plays an important role in regulating the mTORC1 pathway by interacting with Sestrin2, a known inhibitor of mTORC1. This interaction leads to the activation of mTORC1. Once activated, mTORC1 promotes protein synthesis via phosphorylation of eIF4E binding protein 1 (4E-BP1) and ribosomal protein S6 kinase 1 (S6K1). In addition to protein metabolism, BCAAs also contribute to glucose metabolism by improving glycogen uptake and promoting glycogen synthesis in both the liver and skeletal muscle. Regarding the optimal functioning of the immune system, BCAAs support lymphocyte proliferation and stimulate the activity of cytotoxic T cells, primarily through their oxidative breakdown, which is facilitated by the dehydrogenase and decarboxylase enzymes expressed in immune cells [17]. Aromatic amino acids, especially phenylalanine and tyrosine, are found in increased concentrations in obese children and have been linked to the development of metabolic disorders. Trp is the precursor of serotonin (5-hydroxytryptamine), a neurotransmitter known to suppress appetite and promote satiety. Serotonin acts primarily in the hypothalamus to reduce food intake. Increased Trp availability enhances central serotonin synthesis, particularly after carbohydrate intake, due to competitive transport across the blood–brain barrier. Tyrosine, derived from dietary intake or the hydroxylation of phenylalanine, is a precursor for dopamine and norepinephrine. These catecholamines are involved in reward-driven feeding behavior and energy balance, often promoting appetite suppression and increased energy expenditure. Phenylalanine itself may also stimulate satiety hormones such as cholecystokinin (CCK) [18]. The accumulation of these amino acids may reflect disruptions in amino acid catabolic pathways and has been proposed as a potential biomarker indicative of metabolic stress.

The autophosphorylation of tyrosine residues during translation leads to the activation of dual-specificity tyrosine-phosphorylation-regulated kinase 1B (DYRK1B), which subsequently phosphorylates specific serine/threonine residues on its target proteins. This process contributes to hyperlipidemia by activating mTORC2, a regulator of hepatic lipogenesis, and is associated with insulin resistance. Notably, insulin resistance impairs amino acid uptake and utilization in peripheral tissues such as skeletal muscle, leading to elevated circulating amino acid levels, particularly of branched-chain and aromatic amino acids. Furthermore, altered phosphorylation of insulin signaling components may disrupt hepatic amino acid metabolism, reducing catabolic flux through transamination and ureagenesis pathways. As a result, impaired insulin signaling, driven in part by aberrant phosphorylation events, is closely associated with the accumulation of amino acids in the bloodstream, reflecting a broader disturbance in nutrient sensing and metabolic homeostasis [12,13]. In hepatic tissue, insulin signaling is mediated by tyrosine kinase receptors, which help suppress hepatic glucose production, promote glycogen storage, and stimulate fatty acid synthesis, ultimately leading to the formation of triglycerides. Elevated circulating amino acid levels, particularly of BCAAs such as Leu, Ile, and Val, can interfere with insulin signaling by reducing tyrosine phosphorylation of insulin receptor substrates (IRS-1 and IRS-2), thereby contributing to insulin resistance [19].

Although most amino acids are present in elevated concentrations, glycine, serine, and citrulline are often reduced in the plasma of obese individuals. These amino acids play roles in anti-inflammatory processes, oxidative stress control, and endothelial function [20]. Low levels have been correlated with increased insulin resistance and impaired vascular function, suggesting a protective role that may be diminished in obesity [21]. In the context of childhood obesity, disturbances in amino acid metabolism are increasingly recognized as both early markers and contributors to metabolic dysfunction [22,23]. Altered amino acid concentrations, particularly branched-chain and aromatic amino acids, have been associated with insulin resistance, inflammation, and hepatic lipid accumulation in pediatric populations. These alterations not only reflect underlying physiological stress but may also exacerbate disease progression by disrupting implicated pathways such as mTOR signaling, gluconeogenesis, and mitochondrial function. Therefore, amino acid profiling holds promise for improving early detection, risk stratification, and personalized therapeutic strategies in the management of childhood obesity.

## 3. Current Research on Amino Acids and Pediatric Obesity

We investigated the potential role of amino acid interventions in the management of obesity in children, assessing how specific amino acids may affect weight regulation and overall metabolic health. Several recent studies are summarized in Table 1.

Research on the association between amino acids and childhood obesity is still in its early stages, but there are studies that suggest that certain amino acids may influence weight control.

### 3.1. BCAAs and Insulin Resistance

**Figure 2:**
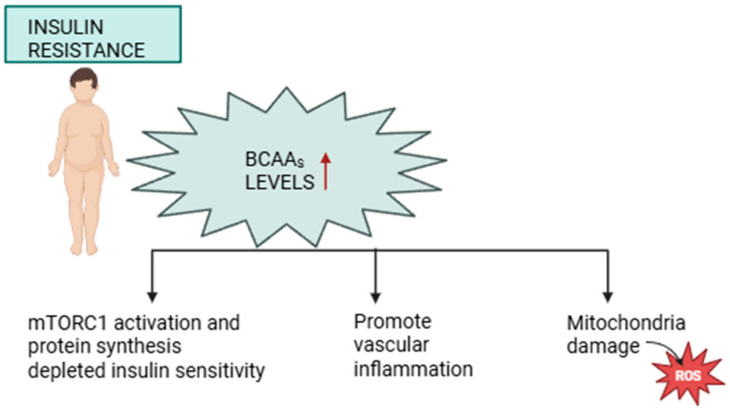
Proposed mechanisms through which elevated BCAAs concentrations contribute to the development of insulin resistance. Created with BioRender.com.

It is not yet very clear whether BCAAs are a causative factor of insulin resistance or a biomarker of impaired insulin action, although there is clear evidence that elevated BCAA concentrations predict future insulin resistance [33]. BCAAs have consistently been shown to be elevated in obese individuals, including children and adolescents. Leu, Ile, and Val are metabolized primarily in skeletal muscle and adipose tissue through the BCAA aminotransferase (EC 2.6.1.42) and branched-chain α-keto acid dehydrogenase complex (BCKDH). Decreased expression or activity of BCKDH in peripheral tissues correlated with obesity results in altered BCAAs catabolism [34]. Obesity is therefore associated with the accumulation of BCAAs and their intermediate metabolites, such as branched-chain α-keto acids. Several mechanisms have been proposed through which increased BCAA concentrations contribute to the development of insulin resistance (Figure 2).

A prominent mechanistic hypothesis implicates the persistent activation of the mTORC1, which regulates protein synthesis but also inhibits insulin signaling when chronically activated [35,36]. Another proposed mechanism is inflammatory signaling. Elevated BCAAs and beta-ketoacid metabolites may stimulate stress kinases, such as Jun *N*-terminal kinase (JNK) and p38 mitogen-activated protein kinase (p38 MAPK), which disrupt insulin receptor signaling [37].

The study by McCormack et al. showed that obesity is linked to increases in BCAAS concentrations in children and adolescents and that these increases can be independently associated with future insulin resistance, as estimated by Homeostatic Model Assessment for Insulin Resistance (HOMA-IR) [38]. The authors considered that the increased catabolic flux of BCAAs could be a consequence of apparent overnutrition early in life, which may result in alterations in insulin action [38]. Similar results were reported by Lee et al. Baseline BCAAs were significantly positively correlated with both HOMA-IR and metabolic risk score. High BCAAs concentrations may be “early” biomarkers for predicting future metabolic diseases [32]. In several studies, increased BCAA concentrations have been correlated with higher levels of the HOMA-IR index [32,39]. Increased intake of BCAAs has increased the risks of obesity and insulin resistance in children, as reported by Lu et al. [40]. Restricting nutritional ingredients such as BCAAs may be important to prevent childhood obesity and insulin resistance [40].

The experimental study by Cojocaru et al. [41] supports the hypothesis that the amino acids Val and Leu exert a protective effect against diet-induced hyperlipidemia. Both amino acids demonstrated a capacity to significantly reduce elevated triacylglycerol levels in rats fed a cholesterol-rich diet [41]. Notably, valine produced a more pronounced and rapid reduction in plasma triacylglycerols compared with leucine. These findings suggest that valine may offer a stronger antiatherogenic benefit, likely through more immediate modulation of lipid metabolism [41]. BCAAs are not the only amino acids that have been investigated as possible markers of obesity and cardiometabolic health. Elevated concentrations of phenylalanine and tyrosine have been found in obese children [29,38,42]. There are studies that have reported opposite results regarding the relationship between BCAAs and obesity. Michaliszyn et al. investigated beta cell function in a cohort of young patients [43]. The authors’ results are opposite to what has been reported before because increased plasma amino acid concentration was positively associated with beta cell function and, consequently, with a lower risk of type 2 diabetes. The results of the study by Hellmuth et al. indicate normal BCAA levels [44]. They conclude that tyrosine alterations in association with insulin resistance precede alterations in BCAA metabolism [44]. However, the change in the ratio of BCAA metabolites may suggest an earlier reduction in BCAA metabolism, which could precede their increase in obese patients and insulin resistance [44,45]. The mechanism through which elevated concentrations of BCAAs in the systemic circulation may cause insulin resistance remains unclear. In fact, it is unclear whether higher concentrations of BCAAs themselves or their disrupted metabolism in various tissues promote insulin resistance.

### 3.2. Amino Acids in Pediatric Metabolic Dysfunction-Associated Steatotic Liver Disease (MASLD)

Metabolic dysfunction-associated steatotic liver disease, previously known as non-alcoholic fatty liver disease (NAFLD), is highly prevalent in obese children and adolescents and represents a serious complication of pediatric obesity. Compared with adults, less is known about MASLD in the pediatric population, with few metabolomic studies published to date.

Plasma amino acid profiling in obese children has revealed characteristic alterations associated with MASLD, independent of body mass index and insulin resistance. Notably, elevated BCAAs are frequently observed [27,46,47,48]. Impaired catabolism of BCAAs, secondary to reduced activity of the branched-chain α-keto acid dehydrogenase complex, leads to systemic accumulation of these metabolites.

A cohort study of 222 patients aged 2–25 years diagnosed with NAFLD aimed to identify a panel of metabolites that could be used as a screening tool for NAFLD. Of the compounds identified, five were assigned to the amino acids Ser, Leu, Ile, and Trp [49]. Goffredo et al. explored whether, independently of obesity and insulin resistance, obese adolescents with NAFLD exhibit a metabolomic signature consistent with abnormalities in amino acid and lipid metabolism [20]. The authors identified elevated levels of Val, Ile, Trp, and Lys in adolescents with NAFLD. They reported that higher baseline valine concentrations were predictive of fat accumulation during a follow-up, suggesting that early alterations in BCAA metabolism may affect the risk of developing or worsening NAFLD [20,45]. Clinically, elevated BCAAs are associated with hepatic insulin resistance, increased hepatic lipogenesis via mTORC1 activation, and enhanced oxidative stress, all contributing to hepatocellular fat accumulation [50,51].

In addition to BCAAs, elevated aromatic amino acids, particularly tyrosine and phenylalanine, have been reported in pediatric MASLD cohorts [26,52,53,54]. These changes may reflect broader disruptions in hepatic and systemic metabolism, including inflammatory signaling and neurotransmitter dysregulation. Importantly, longitudinal studies indicate that baseline elevations in valine can predict increases in hepatic fat content over time, positioning plasma BCAA levels as potential non-invasive biomarkers for disease progression [55,56]. From a diagnostic standpoint, amino acid profiling offers promise for improving risk stratification in children with obesity. Specific metabolomic signatures, characterized by elevated BCAAs and aromatic amino acids combined with reduced glycine and serine concentrations, could enhance early detection of children at risk for MASLD, potentially reducing reliance on invasive liver biopsies or costly imaging modalities. Therapeutically, interventions targeting amino acid metabolism represent an emerging area of interest. Strategies under investigation include dietary modulation of BCAA intake, enhancement of peripheral BCAA oxidation, and pharmacologic inhibition of mTORC1 signaling pathways [13,48,57,58]. However, clinical evidence remains preliminary, and large-scale pediatric trials are necessary to validate the safety, efficacy, and feasibility of these approaches. While current research suggests that plasma amino acid profiles may provide early insight into the progression of MASLD in children and adolescents, inconsistencies between studies and the lack of standardized methodologies highlight the need for further investigation.

### 3.3. Aromatic Amino Acids and Appetite Regulation

In recent years, increasing attention has been directed toward the involvement of aromatic amino acids in the development of obesity, particularly in pediatric populations, where dysregulation of appetite control contributes significantly to excessive weight gain [59,60,61,62].

The amino acid Trp, the precursor of serotonin and the kynurenine pathway, is of particular interest due to its dual role in both appetite regulation and immune modulation. Altered Trp metabolism has been correlated with alterations in satiety signaling, increased inflammation, and altered gut–brain axis [63]. The gut microbiota plays a significant role in modulating the availability and metabolism of Trp. This, in turn, can directly or indirectly influence metabolic homeostasis and appetite regulation. Not only does Trp affect the secretion of gut-derived hormones, but it can also cross the blood–brain barrier to activate central satiety pathways [64]. Studies investigating how dietary Trp levels affect appetite have reported mixed and sometimes contradictory results. Some experimental studies suggest that Trp supplementation increases food intake by stimulating factors such as gastrin, serotonin, neuropeptide Y, and the growth hormone–insulin-like growth factor axis [64,65,66]. In contrast, other studies report that administration of a 5% Trp supplement may promote satiety and reduce food intake in healthy rats [67]. Increasing dietary Trp has been associated with reduced appetite, primarily through enhanced serotonin production in the hypothalamus [68].

The kynurenine pathway is principally regulated at its entry point by the heme dioxygenases indoleamine 2,3-dioxygenase (IDO1/IDO2) and tryptophan 2,3-dioxygenase (TDO). IDO1 expression and activity are markedly upregulated by pro-inflammatory cytokines, particularly interferon-γ (IFN-γ), tumor necrosis factor-α (TNF-α), and interleukin-6 (IL-6), linking immune activation directly to enhanced Trp catabolism. In contrast, hepatic TDO is induced by glucocorticoids and elevated Trp levels, thereby integrating stress and nutritional signals into pathway flux. Chronic low-grade inflammation, as observed in obesity, sustains IDO1 activity in macrophages and dendritic cells, diverting Trp from serotonin synthesis toward kynurenine and its downstream metabolites [69]. The kynurenine pathway, which is often upregulated in inflammatory states, diverts Trp from serotonin production and has been implicated in the development of metabolic syndrome and depressive symptoms, both of which are common comorbidities in pediatric obesity [70].

These contrasting findings suggest that tryptophan’s influence on appetite regulation may reflect a biphasic response, whereby low-to-moderate supplementation supports orexigenic signaling, while high-dose intake facilitates satiety through central serotonergic mechanisms. This highlights the need for further research on how Trp and its metabolites influence appetite and energy balance through the gut–brain axis.

Tyrosine and phenylalanine are precursors for dopamine and norepinephrine, neurotransmitters that modulate reward-related behavior and motivational aspects of eating. Tyr supplementation has been shown to improve cognitive function and may reduce food cravings by enhancing dopaminergic tone [71]. The experimental study conducted by Shipelin et al. shows that Tyr exerts a broader anti-inflammatory and lipid-regulating effect than Trp, which primarily influences energy intake and body weight [72]. Interestingly, while Trp produced a clear anti-obesity effect through weight normalization, it was associated with hepatic lipid accumulation, as evidenced by the presence of large fatty vacuoles. This finding aligns with prior reports that excessive serotonin activity can promote lipogenesis under certain metabolic conditions [73]. Phenylalanine can also indirectly affect appetite by stimulating the release of cholecystokinin (CCK) and glucagon-like peptide-1 (GLP-1) from the gut, hormones known to induce satiety and reduce food intake [73].

Data from pediatric clinical settings remain scarce. A documented randomized, double-blind controlled trial in adolescents with obesity examined protein sources with varying Trp/large neutral amino acids ratios. While the study focused primarily on sleep efficiency, secondary outcomes included appetite and food intake measures; however, the results regarding dietary Trp and energy intake in youth have yet to be published [74].

A recent analysis of the gut microbiota in obese children found significant alterations in Trp-related metabolic pathways, including reduced microbial biosynthesis of tryptophan, phenylalanine, and tyrosine, which may influence appetite and metabolism directly [75]. Another systematic review proposed that changes in tryptophan metabolites, including kynurenines and indoles, are linked to early-life obesity through microbiota-dependent immune and metabolic mechanisms [76].

A controlled study in school-age children (8–12 years) established an average daily Trp requirement of ~4.7 mg/kg, with an upper 95% confidence limit of ~6.1 mg/kg, aligned with current dietary recommendations, demonstrating that safe intake is well below the levels associated with appetite suppression or metabolic changes observed in animal models [77]. Clinical data in children are limited; only preliminary insights from adolescent sleep trials suggest effects on intake, but lack published outcomes. Animal studies show graded and context-dependent effects of dietary Trp on appetite, weight, and metabolic hormones, yet translation to pediatric populations remains uncertain. Safety and requirements in healthy children remain well established at around 5–6 mg/kg/day, with no evidence of adverse appetite effects at standard intake [77].

Understanding how these amino acids influence central and peripheral mechanisms of appetite regulation may open new avenues for therapeutic strategies targeting obesity and its metabolic consequences.

### 3.4. Protective and Anti-Inflammatory Amino Acids

Amino acids are not only structural components of proteins but also act as immunomodulatory agents, influencing inflammation, oxidative stress, and immunity. While certain amino acids, such as branched-chain and aromatic amino acids, are positively associated with obesity and metabolic dysfunction, others appear to exert protective or anti-inflammatory effects [78,79,80,81,82].

In pediatric populations, where the immune system is still maturing, glutamine, arginine, glycine, and serine have anti-inflammatory and protective effects. These effects are particularly relevant in the context of obesity, malnutrition, infections, and critical illness.

Glutamine (Gln) plays a central role in maintaining intestinal barrier integrity and modulating immune responses. In children with severe infections or malnutrition, glutamine supplementation has been associated with reduced intestinal permeability and improved mucosal immunity [83,84,85,86]. Clinical trials have shown that enteral or parenteral glutamine improves outcomes in pediatric intensive care settings, likely by preserving gut integrity and reducing systemic inflammation [87,88,89].

Glycine (Gly), classified as a non-essential amino acid, exhibits notable anti-inflammatory effects by downregulating key pro-inflammatory cytokines such as the tumor necrosis factor-α and interleukin-6 [90,91]. Research using pediatric models of non-alcoholic fatty liver disease has demonstrated that glycine supplementation can help lower liver fat accumulation and boost insulin sensitivity [92,93]. Additionally, glycine contributes to glutathione synthesis and exhibits antioxidant properties, which may suggest potential therapeutic relevance in the context of metabolic inflammation; however, its specific role in pediatric populations remains to be fully elucidated and warrants further investigation [94].

Serine (Ser), although less studied than Gln or Gly, supports redox balance and acts as a precursor for phospholipids and sphingolipids, which are essential for cell membrane integrity. Its role in modulating immune responses is emerging, with some studies indicating that serine availability may influence T cell activation and macrophage polarization [95,96,97]. In pediatric contexts, adequate Ser levels are critical for neural development and may offer protection against neuroinflammatory states [98,99].

Arg is a key substrate for the synthesis of nitric oxide, a molecule vital to maintaining vascular health and facilitating immune communication [100,101]. Studies involving children with burn injuries have shown that arginine supplementation enhances lymphocyte activity, accelerates wound healing, and lowers levels of inflammatory cytokines [13,102,103]. Due to its influence on T-cell behavior and cytokine regulation, arginine holds therapeutic promise for managing immune imbalance and chronic inflammation, particularly in pediatric conditions like obesity [104,105].

While these amino acids show strong therapeutic potential, pediatric-specific dosing and safety data are limited. The majority of existing studies are based on adult populations or focus on critically ill pediatric patients [104,106,107,108,109]. Routine supplementation in pediatric populations, such as those with obesity or low-grade inflammation, requires further study to assess long-term safety, efficacy, and optimal administration routes.

**Figure 3:**
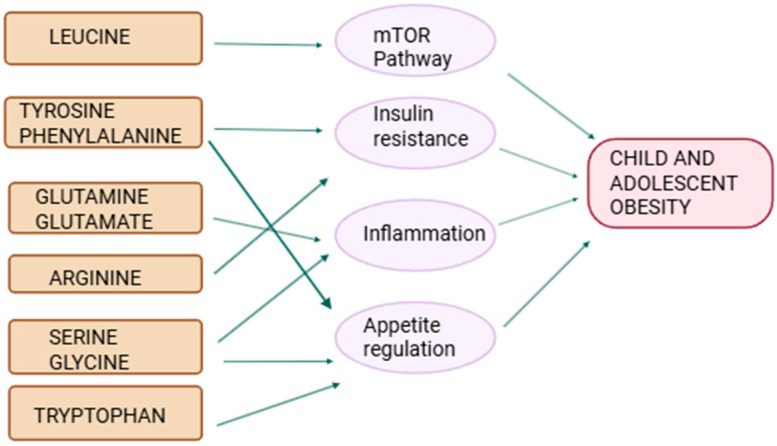
Schematic representation of the relationship between amino acids and pathogenesis of obesity in children and adolescents.

The amino acids such as Gln, Arg, Gly, and Ser contribute to inflammation control and tissue protection in pediatric populations. Their roles in immune regulation, gut barrier integrity, and metabolic balance (Figure 3) make them promising candidates for managing conditions like pediatric obesity, infection, and inflammatory disorders. However, rigorous pediatric trials are essential before these amino acids can be integrated into clinical nutrition protocols for broader pediatric use.

## 4. Conclusions and Future Directions

Amino acids, such as Leu, Val, Trp, Arg, Glu, and Gly, play a significant role in metabolic regulation and appetite control, making them a promising component of childhood obesity management. These amino acids have shown potential in impacting body weight and metabolic health through various pathways, ranging from neurotransmitter synthesis to the modulation of gut-derived hormones and cytokine activity. The current data on the role of amino acids in the management of obesity in children and adolescents suggests that certain amino acid interventions may offer a promising approach to addressing this complex and multifaceted public health issue. Arg has the greatest potential for clinical integration, particularly in cases of serious illness, sickle cell anemia, and oxidative stress. Leu and Trp show promise in more specialized contexts, such as neonatal nutrition, growth disorders, and neurobehavioral conditions. While encouraging initial findings, it is important to note that the majority of available evidence originates from studies conducted on adults or animal models. Research specifically targeting children with obesity remains limited and often lacks consistency in methodology, dosage, and clinical outcomes. This makes drawing definitive conclusions about their effectiveness or safety in pediatric populations difficult. While current research highlights the potential benefits of amino acid modulation, more clinical trials are needed to establish optimal intake levels and long-term safety. Future research should focus on personalized nutritional strategies that incorporate amino acids for effective obesity management. Advancing our understanding of these bioactive nutrients may lead to targeted nutritional interventions that are safe, effective, and tailored to the unique physiological needs of growing children. Incorporating amino acids into pediatric care, when supported by solid evidence, could offer a valuable tool for preventing and managing diseases early in life.

## Figures and Tables

**Table 1:** Summary of the correlation of amino acids with pediatric obesity based on the available literature.

Author, Year, Country	Study Design	Participants	Metabolites	Main Findings
McCann, 2025, USA [24]	Observational Study	220 adolescents aged 10–18 with severe obesity	Serum BCAAs	Obese adolescents had elevated serum BCAAs but reduced beta-ketoacid metabolites compared with healthy subjects.
Garibay-Nieto, N. et al., 2023, Mexico [25]	Cross-sectional comparative study	79 children and adolescents aged 8 to 16 years	Alanine, glycine, leucine, valine, phenylalanine, tyrosine	Glycine and alanine may be the distinguishing variables between metabolic-dysfunction-associated steatotic liver disease patients with and without liver fibrosis.
Chae W. et al., 2022, Republic of Korea [26]	Prospective observational cohort study	165 children and adolescent participants aged 6 to 19 years	Leucine, isoleucine, valine, lysine, tyrosine	Plasma levels of BCAAs, lysine, and tyrosine were significantly elevated in obese children with nonalcoholic fatty liver disease.
Lischka, J. et al., 2021, Austria [27]	Cohort Study	68 patients aged 9–19 years	Alanine, glycine, tyrosine, phenylalanine, BCAAs	Elevated blood levels of BCAAs have been shown to correlate positively with the presence and severity of non-alcoholic fatty liver disease.
Perng W. et al., 2020, USA [28]	Cohort study, Project Viva	524 adolescents aged 13 (13.0 ± 0.7 years)	Valine, 2-methylbutyrylcarnitine, isovaleryl carnitine, propionyl carnitine	Serum concentrations of BCAAs were most elevated in adolescents with concurrent obesity and high metabolic risk compared with those with neither condition.
Perng W. et al., 2019, Mexico [29]	Prospective study in the ELEMENT Project	179 adolescents aged 8–14 years	Alanine, leucine, isoleucine, valine, phenylalanine, tyrosine	Isoleucine, tyrosine, and phenylalanine exhibited strong positive associations with markers of insulin resistance, suggesting their potential role in metabolic dysregulation during puberty.
Goffredo, M. et al., 2017, USA [30]	Prospective observational cohort study	78 children and adolescents aged 8–18 years	Lysine, leucine, isoleucine, valine	Isoleucine and valine have been observed to inversely correlate with insulin sensitivity in peripheral tissues. High BCAAs are linked to impaired hepatic glucose regulation and reduced insulin responsiveness in the liver.
Jin, R. et al., 2016, USA [31]	Prospective case–control study	39 obese adolescents aged 11–17 years	Alanine, glycine, serine, leucine, isoleucine, valine, tyrosine	Among obese adolescents diagnosed with non-alcoholic fatty liver disease, metabolic activity associated with BCAAs, as well as glycine, serine, and alanine, exhibited relatively diminished associations when compared with other metabolic pathways.
Lee A. et al., 2015, Republic of Korea [32]	Cohort Study	109 boys aged 9–11 years	Alanine, glycine, serine, leucine, isoleucine, valine, phenylalanine, tyrosine, lysine	Plasma concentrations of BCAAs are significantly and positively associated with insulin resistance, as estimated by the homeostasis model assessment. In obese pediatric populations, elevated levels of BCAAs, along with the aromatic amino acids phenylalanine and tyrosine, are commonly observed. Conversely, as obesity progresses, glycine and serine levels decrease.

## Data Availability

The authors permit the sharing of all the data.
